# Association between stress hyperglycemia and outcomes in patients with acute ischemic stroke due to large vessel occlusion

**DOI:** 10.1111/cns.14163

**Published:** 2023-03-13

**Authors:** Zhouzhou Peng, Jiaxing Song, Linyu Li, Changwei Guo, Jie Yang, Weilin Kong, Jiacheng Huang, Jinrong Hu, Shuai Liu, Yan Tian, Dahong Yang, Fengli Li, Wenjie Zi, Dongjing Xie, Qingwu Yang

**Affiliations:** ^1^ Department of Neurology, Xinqiao Hospital and The Second Affiliated Hospital Army Medical University Chongqing China

**Keywords:** acute ischemic stroke, endovascular treatment, large vessel occlusion, stress hyperglycemia ratio

## Abstract

**Aims:**

This study aimed to evaluate the association between stress hyperglycemia ratio (SHR) and clinical outcomes at 90 days in acute ischemic stroke due to large vessel occlusion receiving endovascular treatment.

**Methods:**

The RESCUE BT trial was a multicenter, randomized, double‐blind, placebo‐controlled clinical trial, consisting of 948 stroke patients from 55 centers in China. A total of 542 patients with glucose and glycated hemoglobin (HbA1C) values at admission were included in this analysis. SHR, measured by glucose/HbA1C, was evaluated as both a tri‐categorical variable (≤1.07 vs. 1.08–1.29 vs. ≥1.30) and a continuous variable. The primary outcome was a favorable functional outcome (modified Rankin Scale [mRS] score ≤2) at 90 days. The secondary outcome included excellent functional outcome (mRS score ≤1) and safety outcomes, such as 90‐day mortality and intracranial hemorrhage. The study was registered with Chictr.org.cn (ChiCTR‐INR‐17014167).

**Results:**

Compared with patients in the lowest tertile of SHR, the highest tertile group had significantly lower odds of achieving favorable functional outcome of mRS score of 0–2 (adjusted odds ratio, 0.44; 95% confidence interval, 0.28–0.69; *p* < 0.001) and excellent clinical outcome of mRS score of 0–1 (adjusted odds ratio, 0.48; 95% confidence interval, 0.29–0.79; *p* = 0.004) at 90 days after adjusting for potential covariates. Similar results were observed after further adjustment for preexisting diabetes and Alberta Stroke Program Early Computed Tomography Score (ASPECTS).

**Conclusion:**

Stress hyperglycemia ratio, as measured by the glucose/HbA1C, was associated with a decreased odds of achieving a favorable functional outcome in patients with acute large vessel occlusion stroke at 90 days.

## Graphical abstract

Among patients with AIS‐LVO receiving EVT, those in the highest tertiles of SHR have the worst clinical outcomes, suggesting that stress hyperglycemia is associated with poor clinical functional outcome. So early implementation of glucose monitoring may delay stroke progression and improve clinical outcomes after AIS‐LVO.

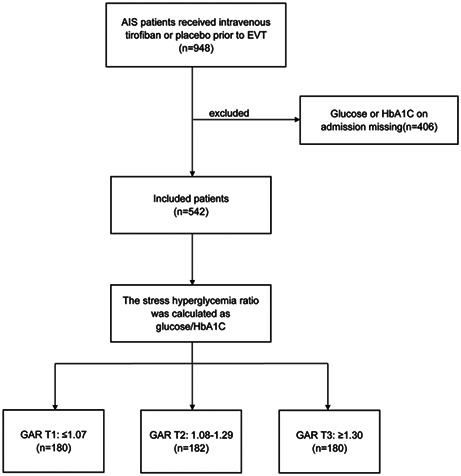

## 1 INTRODUCTION

Acute ischemic stroke (AIS) is a major cause of morbidity and mortality worldwide. Studies have shown that endovascular treatment (EVT) can notably reduce disability at 90 days and improve prognostic functional outcomes in patients with acute stroke due to large vessel occlusion.^1^, ^2^, ^3^

Recently, increasing attention has been paid to the admission glucose level of stroke patients, and there has been significant sound medical evidence that glycemic status should be considered one of the most important modifiable factors for clinical outcomes in patients treated with EVT.^4^, ^5^, ^6^ To a certain extent, early monitoring and management of blood glucose levels can play a key role in the development and prognosis of the disease. In major diseases, such as stroke and myocardial infarction, patients experience a stress response in which plasma glucose levels are often acutely elevated, a phenomenon known as stress hyperglycemia.^7^, ^8^, ^9^ This stressful situation can exacerbate stroke disease progression, subsequently aggravating brain damage to some degree and further increasing the disability and mortality rate.^10^, ^11^, ^12^ However, owing to the influence of various other indicators, using blood glucose or glycated hemoglobin (HbA1C) alone to describe this indicator is insufficient. In previous study Roberts et al. proposed a stress hyperglycemia ratio (SHR) indicator considering both glucose and HbA1C to quantify this stress condition.^10^

Herein, using the data from The Endovascular Treatment With vs. Without Tirofiban for Patients with Large Vessel Occlusion Stroke (RESCUE BT) Trial,^3^ we aimed to evaluate the association between SHR, measured by glucose/HbA1C, and clinical outcomes in patients with acute large vessel occlusion stroke.

## 2 METHODS

### 2.1 Study design and patients selection

The RESCUE BT trial (http://www.chictr.org.cn; ChiCTR‐INR‐17014167) designed to explore the efficacy and safety of intravenous tirofiban in acute ischemic stroke due to large vessel occlusion receiving EVT within 24 h of onset, is a multicenter, randomized, double‐blind, placebo‐controlled clinical trial that enrolled 948 patients with acute large vessel occlusion within 24 h of onset from 55 comprehensive stroke centers in China between October 10, 2018, and October 31, 2021. Eligible patients who had AIS due to anterior circulation large vessel occlusion were randomized to the tirofiban or placebo group at a ratio of 1:1 before endovascular treatment. Meanwhile, all patients were prepared and received endovascular treatment. More details on the protocol and patients' selection criteria of RESCUE BT have been previous published.^3^, ^13^ The ethics committee of the Xinqiao Hospital of the Army Medical University and the research board at each participating center approved the study protocol. All recruited patients or their legal representatives signed a written informed consent form before randomization. In the entire population, 406 cases with missing admission glucose or HbA1C values were excluded from this subgroup analyses. Finally, 542 patients were included in this analysis (Figure S1).

### 2.2 Baseline data collection

Demographic characteristics and baseline clinical characteristics were collected by reviewing patients' electronic medical record file, including sex, age, hypertension, hyperlipidemia, diabetes mellitus, atrial fibrillation, transient ischemic attack, smoking status, blood pressure at admission, National Institutes of Health Stroke Scale (NIHSS) scores at admission, and Alberta Stroke Program Early Computed Tomography Score (ASPECTS) at admission. Laboratory tests (including glucose and HbA1C) and blood pressure measurements were gathered within 24 h of admission. Stroke etiologies were classified according to the Trial of Org 10172 in Acute Stroke Treatment (TOAST). The time from stroke onset to recanalization was recorded.

### 2.3 Assessment of stress hyperglycemia

Relevant data were obtained by reviewing patients' electronic medical records. Blood glucose values and HbA1c levels were measured from serum specimens at the time of admission. Stress hyperglycemia was assessed using the following formula: glucose (mmol/L)/HbA1C (%). The patients were then categorized into three groups and further statistical analyses were performed by tertiles of glucose/HbA1C (T1–T3: ≤1.07 vs. 1.08–1.29 vs. ≥1.30, respectively). This new glucose/HbA1C metric was used to quantify the degree of acute elevation of blood glucose levels in stressful situations.

### 2.4 Study outcomes

The primary efficacy outcome of this analysis was the proportion of favorable functional outcome defined as a modified Rankin Scale (mRS) score of 2 points or less at 90 days. An mRS score of 0–2 was considered a rational outcome in patients with acute large vessel occlusion given its high rates of mortality or disability, as reported previously.^14^, ^15^ Secondary efficacy clinical outcomes included a 90‐day mRS score of 0–1 and a 90‐day distribution of mRS scores. Safety outcomes included 90‐day mortality, the risk of symptomatic intracranial hemorrhage (sICH) classified by Heidelberg, and any intracranial hemorrhage (ICH) within 48 h. All scores were assessed via telephone calls or face‐to‐face interviews by neurologists blinded to the treatment assignment.

### 2.5 Statistical analyses

For continuous variables, the Kolmogorov–Smirnov test was used to test the normality firstly. After the test, all data distributions in this study did not conform to normality. Non‐normally distributed continuous variables and ordinal variables were presented as medians and interquartile ranges (IQRs), and categorical variables were presented as absolute numbers and percentages. When comparing baseline data among glucose/HbA1C ratio at different tertiles, univariate comparisons were performed using Fisher's exact tests or chi‐square test for categorical variables and Kruskal–Wallis test or Mann–Whitney *U*‐test for ordinal or non‐normally distributed continuous variables.

The effects of different SHR values on functional outcomes (mRS score of 0–2 and 0–1) and safety outcomes (mortality and intracranial hemorrhage) were assessed using a binary logistic regression model. In the multivariable logistic regression analysis, two models were included in the analysis. The final factors incorporated included those with significance (*p* < 0.1) across tertiles of SHR and those associated with poor functional outcomes in previous studies. In the first model, we adjusted variables including age, sex, smoking, hypertension, hyperlipidemia, baseline NIHSS score, occlusion site, and stroke etiology. In another model, we further adjusted for preexisting diabetes and baseline ASPECTS. Ordinal logistic regression analysis and nonparametric Kruskal–Wallis tests were used to identify differences in the distribution of mRS grades among patients across tertiles of SHR. Subsequently, subgroup analyses were conducted to examine the consistency between SHR and favorable functional outcome in patients with different baseline characteristics. The odds ratios (OR) with 95% confidence intervals (CIs) are also reported in each table. In addition, we employed receiver operating characteristic (ROC) curves to investigate the ideal cutoff values of SHR to distinguish clinical functional outcomes. Furthermore, we analyzed the association between SHR and the favorable outcome and mortality using adjusted margin plots. Restricted cubic splines were also plotted to find the association between SHR level and clinical outcomes.

All statistical analyses were performed using SPSS Statistics version 26 (IBM Corp.) and R. version 4.0.5 (https://www.r‐project.org). Results were considered statistically significant at two‐tailed *p* < 0.05. We excluded patients with missing essential data from our analysis.

## 3 RESULTS

### 3.1 Study participants and baseline characteristics

A total of 948 patients with acute large vessel occlusion stroke were enrolled from the 55 sites in the RESCUE BT trial, among which 406 patients had missing admission glucose values and were therefore excluded from this analysis. The baseline characteristics of the patients included and excluded are summarized in Table S1. Finally, 542 patients were included in this analysis, including 236 women (43.5%) and 306 men (56.5%). The median (IQR) age, baseline NIHSS score, and baseline ASPECTS were 68 (58–75) years, 16 (12–19), and 8 (7–9), respectively. Table 1 shows the baseline characteristics of the patients included by tertiles of SHR.

**TABLE 1:** Baseline characteristic of the subgroups of trichotomized stress hyperglycemia ratio values

Characteristics	All patients	Tertile 1 (≤1.07)	Tertile 2 (1.08–1.29)	Tertile 3 (≥1.30)	*p*‐value
Number of patients	542	180	182	180	
Age, median (IQR), years	68 (58–75)	68 (57–74)	68 (56–75)	70 (61–76)	0.174
Sex, female, No./Total No. (%)	236/542 (43.5)	64/180 (35.6)	75/182 (41.2)	97/180 (53.9)	0.002
Medical history, No./Total No. (%)					
Hypertension	311/542 (57.4)	100/180 (55.6)	105/182 (57.7)	106/180 (58.9)	0.811
Hyperlipidemia	82/542 (15.1)	26/180 (14.4)	23/182 (12.6)	33/180 (18.3)	0.303
Diabetes mellitus	150/542 (27.7)	36/180 (20.0)	36/182 (19.8)	78/180 (43.3)	<0.001
Smoking	106/542 (19.6)	36/180 (20.0)	44/182 (24.2)	26/180 (14.4)	0.065
Atrial fibrillation	186/542 (34.3)	63/180 (35.0)	58/182 (31.9)	65/180 (36.1)	0.678
Transient ischemic attack	7/542 (1.3)	4/180 (2.2)	1/182 (0.5)	2/180 (1.1)	0.322
SBP, median (IQR), mmHg	145 (129–162)	141 (124–158)	145 (128–160)	149 (134–170)	0.006
Glucose, median (IQR), mmol/liter	6.92 (5.77–8.74)	5.46 (4.96–5.89)	6.89 (6.28–7.44)	9.64 (8.16–12.30)	<0.001
HbA1C, median (IQR), %	5.90 (5.50–6.46)	5.80 (5.60–6.25)	5.80 (5.43–6.20)	6.10 (5.60–7.40)	0.002
SHR, median (IQR)	1.17 (1.00–1.37)	0.95 (0.85–1.00)	1.17 (1.11–1.23)	1.53 (1.37–1.72)	
Baseline NIHSS score, median (IQR)	16 (12–19)	16 (11–19)	16 (12–19)	16 (12–20)	0.205
Baseline ASPECTS, median (IQR)	8 (7–9)	8 (7–9)	8 (7–9)	7 (6–9)	0.047
Stroke etiology, No./Total No. (%)					
LAA	246/542 (45.4)	81/180 (45.0)	79/182 (43.4)	86/180 (47.8)	0.664
CE	240/542 (44.3)	77/180 (42.8)	83/182 (45.6)	80/180 (44.4)	
Other causes	56/542 (10.3)	22/180 (12.2)	20/182 (11.0)	14/180 (7.8)	
Occlusion site, No./Total No. (%)					
ICA intracranial	118/542 (21.8)	42/180 (23.3)	39/182 (21.4)	37/180 (20.6)	0.746
MCA‐M1	346/542 (63.8)	109/180 (60.6)	116/182 (63.7)	121/180 (67.2)	
MCA‐M2	78/542 (14.4)	29/180 (16.1)	27/182 (14.8)	22/180 (12.2)	
Group, No./Total No. (%)					
Tirofiban	280/542 (51.7)	94/180 (52.2)	91/182 (50.0)	95/180 (52.8)	0.855
Placebo	262/542 (48.3)	86/180 (47.8)	91/182 (50.0)	85/180 (47.2)	
Onset to recanalization, median (IQR), min	464 (316–696)	490 (310–754)	475 (329–686)	452 (309–669)	0.572

*Abbreviations*: ASPECTS, Alberta Stroke Program Early Computed Tomography Score; CE, cardio‐embolism; HbA1C, glycated hemoglobin; ICA, internal carotid artery; IQR, interquartile range; LAA, large artery atherosclerosis; MCA, middle cerebral artery; NIHSS, National Institutes of Health Stroke Scale; SBP, systolic blood pressure; SHR, stress hyperglycemia ratio.

In these three groups, 180 patients had an SHR ≤1.07, 182 patients had an SHR value of 1.08–1.29, and 180 patients had an SHR value ≥1.30. As shown, patients with lower SHR had lower glucose (5.46 [4.96–5.89] vs. 6.89 [6.28–7.44] vs. 9.64 [8.16–12.30], *p* < 0.001), lower HbA1c (5.80 [5.60–6.25] vs. 5.80 [5.43–6.20] vs. 6.10 [5.60–7.40], *p* = 0.002), and lower systolic blood pressure (141 [124–158] vs. 145 [128–160] vs. 149 [134–170] for SHR≤1.07 vs. 1.08–1.29 vs. ≥1.30, respectively, *p* = 0.006).

### 3.2 Primary outcome

As shown, the higher the SHR of a patient, the lower the probability of achieving a favorable functional outcome (T3 vs. T1: OR = 0.44; 95% CI, 0.28–0.69; *p* < 0.001) in adjusted model 1 (Table 2). The association between tertiles of SHR and outcomes remained statistically significant after further adjustment for preexisting diabetes and baseline ASPECTS (T3 vs. T1: OR = 0.49; 95% CI, 0.31–0.79; *p* = 0.003) in model 2. In addition, we plotted a restrictive cubic spline (Figure S2a), to graphically illustrate the association between SHR and favorable functional outcome (adjusted *p* for non‐linearity = 0.34). Furthermore, as illustrated in the Figure S2c, the probability of estimating a favorable functional outcome decreased as the SHR value increased.

**TABLE 2:** Efficacy outcomes and safety outcomes

Outcome	SHR levels	Events, *n* (%)	*p*‐value	Crude Model		Model 1^a^		Model 2^b^	
				Unadjusted OR (95% CI)	*p*‐value	Adjusted OR (95% CI)	*p*‐value	Adjusted OR (95% CI)	*p*‐value
mRS 0–2	Tertile 1 (≤1.07)	103 (57.2)	<0.001	Reference		Reference		Reference	
	Tertile 2 (1.08–1.29)	91 (50.0)		0.75 (0.49–1.13)	0.169	0.77 (0.50–1.19)	0.242	0.74 (0.48–1.16)	0.188
	Tertile 3 (≥1.30)	62 (34.4)		0.39 (0.26–0.60)	<0.001	0.44 (0.28–0.69)	<0.001	0.49 (0.31–0.79)	0.003
mRS 0–1	Tertile 1 (≤1.07)	73 (40.6)	0.001	Reference		Reference		Reference	
	Tertile 2 (1.08–1.29)	63 (34.6)		0.78 (0.51–1.19)	0.244	0.80 (0.51–1.27)	0.348	0.76 (0.47–1.21)	0.241
	Tertile 3 (≥1.30	41 (22.8)		0.43 (0.27–0.68)	<0.001	0.48 (0.29–0.79)	0.004	0.53 (0.32–0.89)	0.016
Mortality	Tertile 1 (≤1.07)	26 (14.4)	0.037	Reference		Reference		Reference	
	Tertile 2 (1.08–1.29)	21 (11.5)		0.77 (0.42–1.43)	0.412	0.68 (0.36–1.31)	0.252	0.70 (0.36–1.35)	0.287
	Tertile 3 (≥1.30)	38 (21.1)		1.59 (0.92–2.74)	0.100	1.34 (0.75–2.42)	0.325	1.13 (0.61–2.07)	0.703
HBC_sICH	Tertile 1 (≤1.07)	10 (5.6)	0.102	Reference		Reference		Reference	
	Tertile 2 (1.08–1.29)	10 (5.5)		0.99 (0.40–2.44)	0.980	0.97 (0.39–2.41)	0.943	0.99 (0.39–2.49)	0.983
	Tertile 3 (≥1.30)	19 (10.6)		2.01 (0.91–4.45)	0.086	1.95 (0.86–4.42)	0.112	1.85 (0.79–4.32)	0.157
Any ICH within 48 h	Tertile 1 (≤1.07)	51 (28.3)	0.361	Reference		Reference		Reference	
	Tertile 2 (1.08–1.29)	52 (28.6)		1.01 (0.64–1.60)	0.960	1.03 (0.64–1.64)	0.912	1.09 (0.68–1.75)	0.734
	Tertile 3 (≥1.30)	62 (34.4)		1.33 (0.85–2.08)	0.212	1.32 (0.83–2.10)	0.242	1.12 (0.69–1.82)	0.641

*Abbreviations*: ASPECTS, Alberta Stroke Program Early Computed Tomography Score; CI, confidence interval; HBC_sICH, Heidelberg bleeding classification symptomatic intracranial hemorrhage; ICH, intracranial hemorrhage; mRS, modified Rankin Scale; NIHSS, National Institutes of Health Stroke Scale; OR, odds ratio.

^a^ Model 1 adjusted for age, sex, smoking, hypertension, hyperlipidemia, baseline NIHSS score, occlusion site, and stroke etiology.

^b^ Model 2 adjusted for model 1 + history of diabetes mellitus and baseline ASPECTS.

### 3.3 Secondary outcomes

**FIGURE 1:**
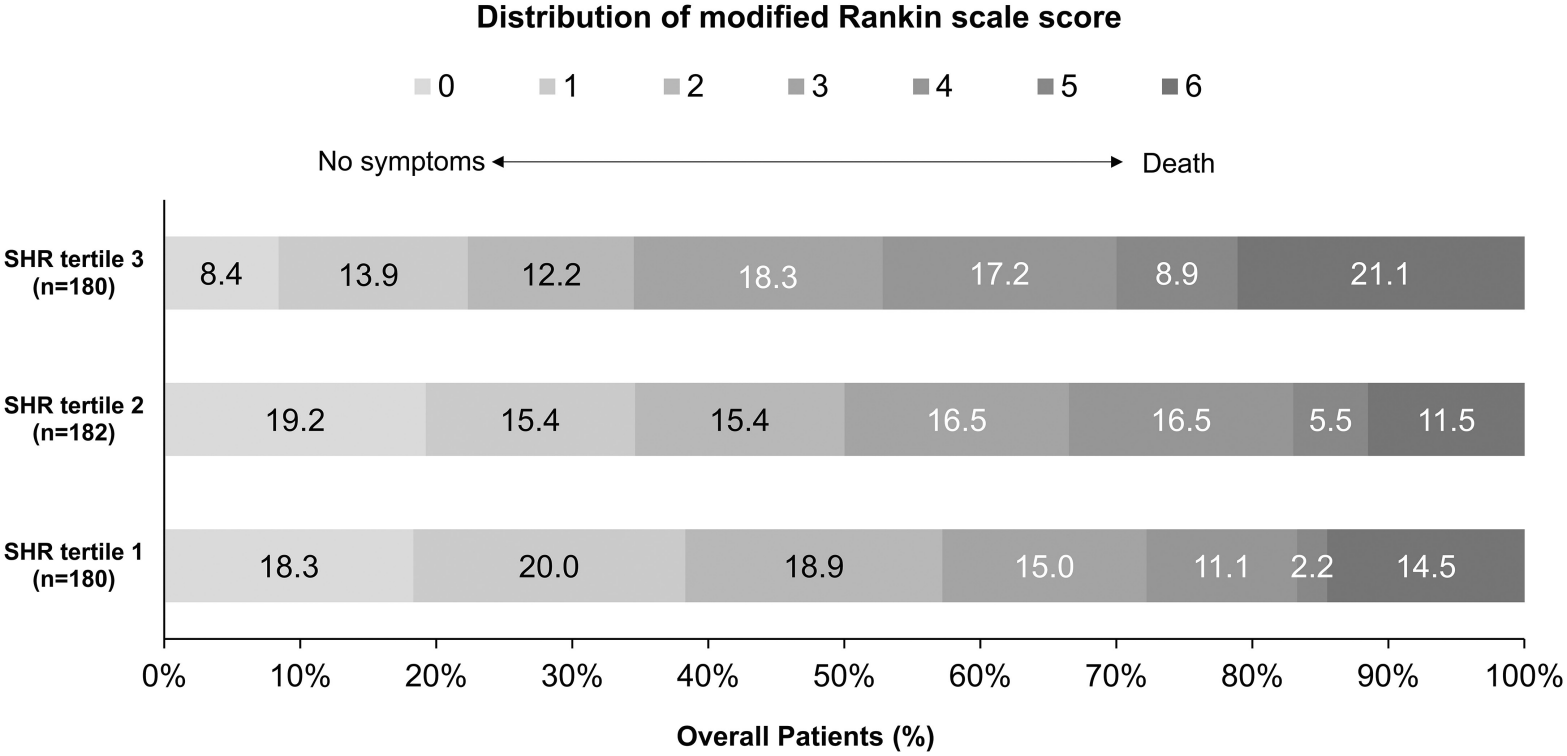
Distribution of the modified Rankin Scale (mRS) scores at 90 days. The distributions of the mRS score among patients with large vessel occlusion were presented according to the trichotomized SHR (*p* < 0.001 by Kruskal–Wallis test). Bars are labelled with proportions. SHR indicates stress hyperglycemia ratio

Secondary efficacy outcomes in this analysis included 90‐day mRS score of 0–1, and 90‐day distribution of mRS scores. As presented in Table 2, compared with patients in the lowest tertiles, patients in the highest tertiles of SHR had a 0.48‐fold probability of achieving an excellent functional outcome at 3 months (22.8% vs. 40.6%; unadjusted OR, 0.48; 95% CI, 0.29–0.79; *p* = 0.004). Similar results for this association were observed after further adjustment in model 2 (T3 vs. T1: adjusted OR, 0.53; 95% CI, 0.32–0.89; *p* = 0.016). However, no statistically significant differences between tertiles of the SHR and safety outcomes, including mortality at 90 days, risk of symptomatic intracranial hemorrhage classified by Heidelberg and any ICH within 48 h. Additionally, the distribution of the mRS scores at 90 days was also presented in Figure 1.

### 3.4 Predictive values of glucose, HbA1c, and stress hyperglycemia ratio for clinical outcomes

We used receiver operating characteristic (ROC) curves to assess the predictive value of glucose, HbA1c, and SHR for clinical outcomes after AIS (Figure S3). Clinical outcome indicators included favorable functional outcomes (mRS score of 0–2), excellent functional outcomes (mRS score of 0–1), and mortality at 90 days. Simultaneously, the area under the curve for SHR was 0.60, 0.58, and 0.58 for favorable functional outcome, excellent functional outcome, and 90‐day mortality, respectively (Table 3). The *p*‐values of the SHR‐based predictors of clinical outcomes were all <0.05. In addition, the ideal cutoff values of SHR were 1.303 with a sensitivity and specificity of 77.0 and 40.6, respectively, for obtaining a favorable outcome and 1.314 with a sensitivity and specificity of 79.1 and 35.9, respectively, for achieving an excellent outcome, both of which indicate that SHR is a reasonable predictor.

**TABLE 3:** Clinical outcome at 90 days after acute large vessel occlusion stroke according to SHR, glucose and HbA1C parameters

Parameters	Cut‐off	AUC (95% CI)	Sensitivity	Specificity	Youden Index	*p*‐value	ROC curve comparison^a^	Adjusted OR^b^ (95% CI)	*p*‐value
Favorable outcome									
SHR	1.303	0.60 (0.55–0.65)	77.0	40.6	0.175	<0.001	Reference	0.47 (0.27–0.82)	0.008
Glucose	7.565	0.61 (0.56–0.66)	70.7	46.5	0.172	<0.001	0.368	0.90 (0.85–0.96)	0.001
HbA1C	6.285	0.55 (0.50–0.60)	75.0	36.7	0.117	0.042	0.155	0.88 (0.77–1.00)	0.053
Excellent outcome									
SHR	1.314	0.58 (0.53–0.63)	79.1	35.9	0.150	0.003	Reference	0.69 (0.38–1.23)	0.204
Glucose	7.680	0.58 (0.53–0.63)	72.3	41.4	0.137	0.002	0.880	0.94 (0.88–1.00)	0.060
HbA1C	6.220	0.54 (0.49–0.59)	75.7	35.1	0.108	0.153	0.244	0.92 (0.79–1.06)	0.246
Mortality									
SHR	1.424	0.58 (0.50–0.65)	37.6	81.4	0.190	0.025	Reference	2.48 (1.35–4.57)	0.004
Glucose	7.565	0.59 (0.52–0.66)	54.1	64.6	0.187	0.011	0.556	1.10 (1.03–1.18)	0.007
HbA1C	6.285	0.56 (0.49–0.63)	43.5	71.1	0.146	0.086	0.715	1.10 (0.92–1.30)	0.294

*Abbreviations*: AUC, area under the curve; CI, confidence interval; HbA1c, glycated hemoglobin; NIHSS, National Institutes of Health Stroke Scale; OR, odds ratio; ROC, receiver operating characteristic; SHR, stress hyperglycemia ratio.

^a^ DeLong test *p*‐value.

^b^ The multiple logistic regression test was used to analyze ORs. Adjusted variables: age, sex, smoking, hypertension, hyperlipidemia, baseline NIHSS sore, occlusion site, and stroke etiology.

### 3.5 Subgroup analyses

**FIGURE 2:**
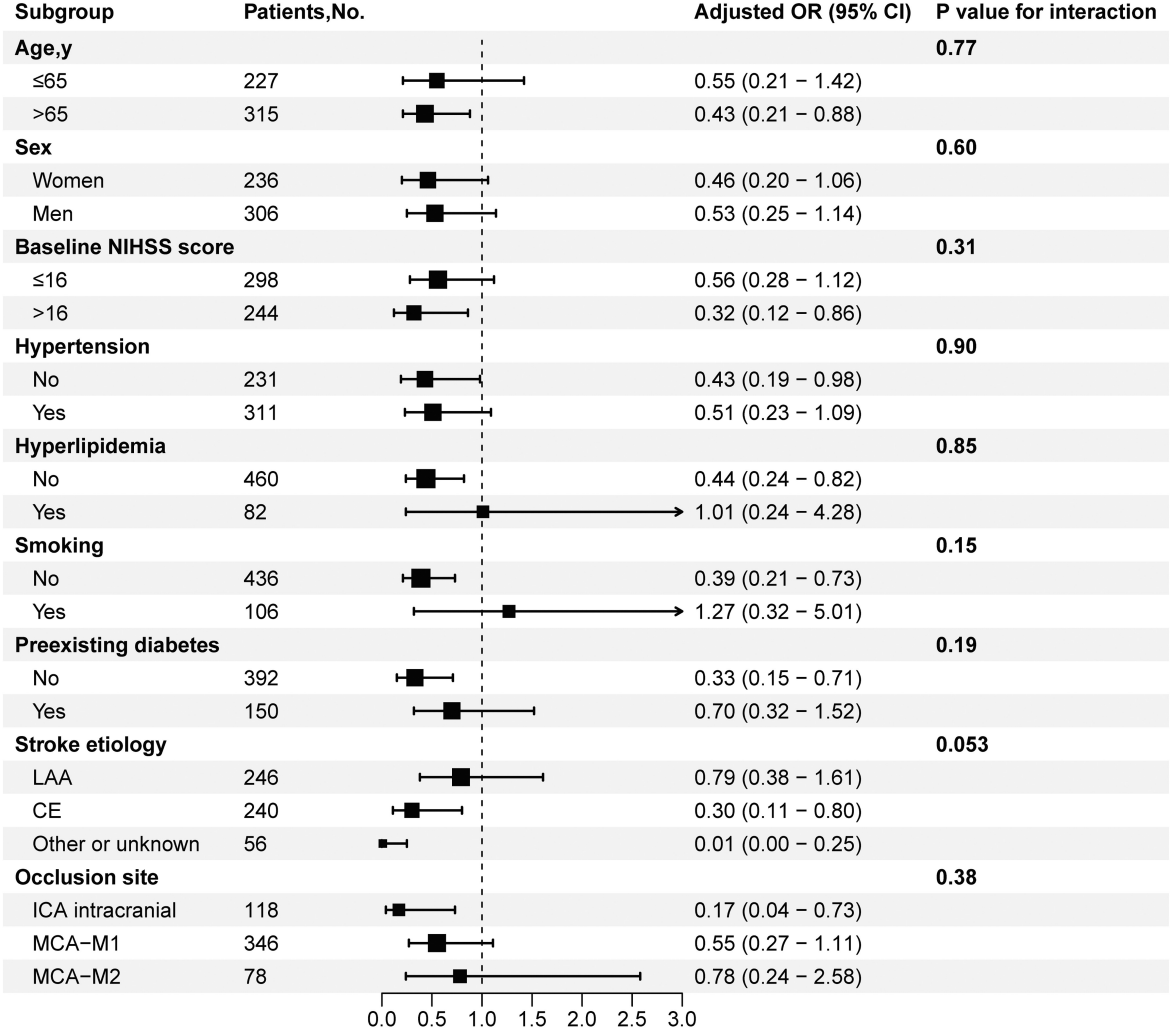
Subgroup analyses of clinical outcomes. Forest plots show that the difference in correlation between stress hyperglycemia ratio and favorable outcome in subgroups of patients with different characteristics at 90 days. Adjusted variables include age, sex, smoking history, baseline National Institutes of Health Stroke Scale (NIHSS) score, hypertension, hyperlipidemia, preexisting diabetes, stroke etiology and occlusion site. LAA, large artery atherosclerosis; CE, cardio‐embolism; ICA, internal carotid artery; MCA, middle cerebral artery

We performed subgroup analyses to further explore the association between SHR values and primary outcome in different subpopulations. There was a consistent effect of SHR on 90‐day favorable outcome across all subgroups and interaction analysis also showed no heterogeneity among patients with different baseline characteristics (*p* for interaction >0.05, Figure 2). Additionally, SHR was associated with unfavorable outcome in tirofiban group (T3 vs. T1: adjusted OR, 0.36; 95% CI, 0.18–0.69, Tables S2 and S3).

## 4 DISCUSSION

In this study, we evaluated the association between the stress hyperglycemia ratio and clinical outcomes in patients with acute large vessel occlusion stroke receiving endovascular treatment. Patients received intravenous tirofiban or placebo prior to EVT. We found that, on admission, higher stress hyperglycemia of stroke patients was closely linked to unfavorable clinical outcomes. However, the association between SHR and safety outcomes was not elucidated.

Patients with acute stroke or myocardial infarction have a high probability of developing stress hyperglycemia during their illness.^4^, ^7^, ^9^ Studies over the past two decades have focused on the correlation between stress hyperglycemia, disability, and mortality after stroke.^4^, ^16^, ^17^ There already existed reliable medical evidence which showed higher admission glucose levels in non‐survivors of stroke than in AIS survivors.^7^ Two large studies also elucidated that glucose level at admission is a meaningful independent predictor of mortality or poor functional prognosis after stroke.^18^, ^19^ In addition, researchers have focused on management strategies and treatment modalities for post‐stroke hyperglycemia and have explored the association between the intensive management of hyperglycemia with insulin‐based regimens and clinical outcomes, which suggests that this emergency condition has gained the attention of investigators.^20^ In our findings, SHR, calculated as glucose/HbA1C, is associated with unfavorable functional outcome. That is, the higher the SHR of the stroke population, the worse the neurological recovery effect. Using the data from the RESCUE BT trial, which has multicenter and large sample size strengths, our conclusions are consistent with some previous studies, further validating the existing medical evidence.^6^, ^14^

Although this mechanism is not yet fully explored, there are several explanations for this association between stress hyperglycemia and an increased risk of adverse clinical outcomes after stroke. Firstly, higher stress hyperglycemia values may imply a greater stressful inflammatory response to acute critical illness, which promotes the progression of neuroinflammation and release of neurotoxic and vasoconstrictive factors that further damage vascular endothelial cells, leading to a reduction in vascular repair and protection.^10^ There is evidence that acute fluctuations in glucose concentrations in critical illness settings increase patient mortality, and this association is independent of mean glucose concentrations.^12^, ^21^ Repetitive acute glucose fluctuations induce more endothelial apoptosis, greater endothelial dysfunction, and an oxidative stress response cascade.^22^, ^23^ This can deteriorate the progression of stroke to an extent. Second, stress hyperglycemia may directly poison ischemic brain tissue. It has been reported that a large amount of lactic acid produced by anaerobic metabolism of glucose in hyperglycemic patients can accumulate in the brain, leading to intracellular acidosis, which may further accelerate ischemic brain injury by enhancing the effects of lipid and free radical peroxidation.^24^ In an animal model of stroke, hyperglycemia accelerates symptoms of cellular acidosis in the ischemic penumbra, resulting in larger infarct size than in hypoglycemic animals treated with insulin.^25^ In addition, another experiment has also shown that acute post‐stroke hyperglycemia could impair cortical collateral perfusion and further exacerbate stroke injury in rat model, which may in part explain the worse neurological recovery and functional outcome after stroke in the presence of acute hyperglycemia.^26^ Third, stress hyperglycemia may lead to abnormal aggregation of platelets. Previous studies have shown that stress hyperglycemia adversely affects platelet function in patients with acute coronary syndrome (ACS), further exacerbating the condition. Conversely, decreased platelet aggregability has been reported in association with improved diabetic control and blood thrombogenicity.^27^ Therefore, this may also be the potential mechanism of damage in stroke patients with stress hyperglycemia. Fourth, hyperglycemia may damage the blood–brain barrier and promote the transformation of hemorrhagic infarction.^28^ Previous studies have confirmed that stress hyperglycemia is associated with an increased risk of stroke in patients with minor ischemic stroke or transient ischemic attack.^4^ A recent study also showed that SHR was an independent predictor of the risk of symptomatic intracranial hemorrhage in stroke patients, thus worsening their clinical outcomes.^29^ However, the correlation between stress hyperglycemia and the safety outcome indicators of patients was not statistically significant in our study. These associations require further investigation in the future.

Stress hyperglycemia is strictly defined as hyperglycemia at the time of admission in patients without previous diabetes or worsening hyperglycemia in patients with diabetes and is an emergency response to a stressful event.^8^ However, in actual clinical situations, due to the confounding influence of many other factors, it is difficult to define stress hyperglycemia in the entire population by the blood glucose value on admission alone. HbA1c is a measure of blood glucose levels over the past 8–12 weeks and provides an indication of the estimated average blood glucose concentration level over this period, it is often used to determine treatment adequacy and guide adjustment and, thus, can better serve as an indicator of background blood glucose.^30^ Therefore, in this study, we used HbA1c to calculate background glucose and its relationship to admission glucose, calculated as the glucose/HbA1C, to better identify and quantify stress hyperglycemia. Early identification of stress hyperglycemia (relative hyperglycemia) and implementation of relevant interventions may improve patient prognosis and prevent further disease progression, thereby reducing mortality and disability rates.

Our study has some limitations. Firstly, since our study was a post hoc analysis of the RESCUE BT trial, excluding patients without admission glucose and HbA1C values from this study, there may exist selection bias due to the inherent limitation of the study design. Secondly, our stroke population was screened from multiple centers in China, the incidence of stroke in Western populations remains unknown. Thus, further research incorporating other populations is needed in the future to achieve generalizability.

## 5 CONCLUSION

Our results demonstrated that, stress hyperglycemia ratio, as measured by the glucose/ HbA1C, was associated with a decreased odds of achieving a favorable functional outcome in patients with acute large vessel occlusion stroke at 90 days. Therefore, early recognition of such emergency state and implementation of clinical interventions are essential to improve clinical prognosis.

## AUTHOR CONTRIBUTIONS

ZP, JS, and LL contribute equally to the manuscript. ZP, JS, and LL interpreted the data and drafted the manuscript. DX and QY contributed to the conception and design of the study. ZP, JS, and CG did the statistical analyses and creation of figures and tables. ZP, JS, LL, CG, JY, WK, FL, JH, SL, YT, DY, and WZ completed data collection. WZ, DX, and QY provided technical or material support and made critical revision of the manuscript.

## FUNDING INFORMATION

This work was supported by the Army Medical University Xinqiao Hospital Discipline Talent Construction Special Project (NO.2022XKRC003), Science‐health Joint Medical Scientific Research Project of Chongqing (NO.2020FYYX206) and Chongqing major disease control technology research project (NO.2019ZX001).

## CONFLICT OF INTEREST STATEMENT

The authors declare that they have no competing interests.

## Supporting information


Appendix S1.

## Data Availability

The datasets analyzed during the study are available from the corresponding author on reasonable request.
