# Supplementary material for: Association between stress hyperglycemia and outcomes in patients with acute ischemic stroke due to large vessel occlusion
Source: CNS Neurosci Ther. 2023 Mar 13;29(8):2162–70. doi: 10.1111/cns.14163 (PMC10352867; doi:10.1111/cns.14163)
Supplement: Supplementary file 1 — Appendix S1. [file CNS-29-2162-s001.pdf]

Association between stress hyperglycemia and outcomes in patients with acute ischemic stroke due to large vessel occlusion

Supplementary materials

This appendix has been provided by the authors to give readers additional information about their work.

1.Supplemental tables

1.1 eTable 1 Baseline characteristic of patients included and excluded

**Characteristics**	**All (n= 948)**	**Included (n= 542)**	**Excluded (n= 406)**	**P value**
Age, median (IQR), years	67 (57–74)	68 (58–75)	66 (57–74)	0.105
Sex, female, No./Total No. (%)	391/948 (41.2)	236/542 (43.5)	155/406 (38.2)	0.097
**Medical history, No./Total No. (%)**				
Hypertension	524/948 (55.3)	311/542 (57.4)	213/406 (52.5)	0.132
Hyperlipidemia	132/948 (13.9)	82/542 (15.1)	50/406 (12.3)	0.216
Diabetes mellitus	204/948 (21.5)	150/542 (27.7)	54/406 (13.3)	<0.001
Smoking	222/948 (23.4)	106/542 (19.6)	116/406 (28.6)	0.001
Atrial fibrillation	313/948 (33.0)	186/542 (34.3)	127/406 (31.3)	0.325
Transient ischemic attack	10/948 (1.1)	7/542 (1.3)	3/406 (0.7)	0.615
SBP, median (IQR), mmHg	145.00 (130.00–160.00)	145.00 (129.00–162.00)	145.50 (130.00–160.00)	0.615
Glucose, median (IQR), mmol/liter	6.91 (5.75–8.62)	6.92 (5.77–8.74)	6.90 (5.70–8.49)	0.480
HbA1C, median (IQR), %	5.90 (5.50–6.42)	5.90 (5.50–6.46)	5.95 (5.38–6.40)	0.909

SHR, median (IQR)	1.17 (1.00–1.37)	1.17 (1.00–1.37)	NA	NA
Baseline NIHSS score, median (IQR)	16.00 (12.00–19.00)	16.00 (12.00–19.00)	16.00 (12.00–19.00)	0.997
Baseline ASPECTS, median (IQR)	8.00 (7.00–9.00)	8.00 (7.00–9.00)	8.00 (7.00–9.00)	0.669
**Stroke etiology, No./Total No. (%)**				0.424
LAA	435/948 (45.9)	246/542 (45.4)	189/406 (46.6)	
CE	406/948 (42.8)	240/542 (44.3)	166/406 (40.9)	
Other causes	107/948 (11.3)	56/542 (10.3)	51/406 (12.6)	
**Occlusion sites, No./Total No. (%)**				0.514
ICA intracranial	194/948 (20.5)	118/542 (21.8)	76/406 (18.7)	
MCA-M1	615/948 (64.9)	346/542 (63.8)	269/406 (66.3)	
MCA-M2	139/948 (14.7)	78/542 (14.4)	61/406 (15.0)	
**Group, No./Total No. (%)**				0.045
Tirofiban	463/948 (48.8)	280/542 (51.7)	183/406 (45.1)	
Placebo	485/948 (51.2)	262/542 (48.3)	223/406 (54.9)	
Onset to recanalization, median (IQR), min	481.50 (326.50–725.25)	464.00 (315.75–696.25)	511.50 (333.75–763.50)	0.049

Abbreviation: IQR, interquartile range; SBP, systolic blood pressure; HbA1C, glycated hemoglobin; SHR, stress hyperglycemia ratio; NIHSS, National Institutes of Health Stroke Scale; ASPECTS, Alberta Stroke Program Early Computed Tomography Score; LAA, large artery atherosclerosis; CE, cardio-embolism; ICA, internal carotid artery; MCA, middle cerebral artery.

1.2 eTable 2 Baseline characteristics stratified by SHR tertiles in different clinical treatment group

	Placebo					Tirofiban				
	All	Tertile 1 (≤1.07)	Tertile 2 (1.08-1.29)	Tertile 3 (≥1.30)	P value	All	Tertile 1 (<1.07)	Tertile 2 (1.08-1.29)	Tertile 3 (≥1.30)	P value
**Number of patients**	262	86	91	85		280	94	91	95	
**Demographic characteristics**										
Age, median (IQR), years	68 (57–75)	68 (58–74)	67 (55–73)	69 (60–77)	0.192	69 (58–75)	68 (56–74)	69 (57–76)	70 (62–76)	0.321
Female, No./Total No. (%)	106/262 (40.5)	35/86 (40.7)	31/91 (34.1)	40/85 (47.1)	0.214	130/280 (46.4)	29/94 (30.9)	44/91 (48.4)	57/95 (60.0)	<0.001
**Medical history, No./Total No. (%)**										
Hypertension	151/262 (57.6)	49/86 (57.0)	52/91 (57.1)	50/85 (58.8)	0.964	160/280 (57.1)	51/94 (54.3)	53/91 (58.2)	56/95 (58.9)	0.782
Hyperlipidemia	29/262 (11.1)	11/86 (12.8)	8/91 (8.8)	10/85 (11.8)	0.677	53/280 (18.9)	15/94 (16.0)	15/91 (16.5)	23/95 (24.2)	0.269
Diabetes mellitus	76/262 (29.0)	19/86 (22.1)	20/91 (22.0)	37/85 (43.5)	0.002	74/280 (26.4)	17/94 (18.1)	16/91 (17.6)	41/95 (43.2)	<0.001
Smoking	57/262 (21.8)	19/86 (22.1)	27/91 (29.7)	11/85 (12.9)	0.027	49/280 (17.5)	17/94 (18.1)	17/91 (18.7)	15/95 (15.8)	0.860
Atrial fibrillation	79/262 (30.2)	26/86 (30.2)	27/91 (29.7)	26/85 (30.6)	0.991	107/280 (38.2)	37/94 (39.4)	31/91 (34.1)	39/95 (41.1)	0.595
Transient ischemic attack	3/262 (1.1)	1/86 (1.2)	1/91 (1.1)	1/85 (1.2)	>0.999	4/280 (1.4)	3/94 (3.2)	NA	1/95 (1.1)	0.277

SBP, median (IQR), mmHg	145 (128–161)	144 (124–160)	142 (128–160)	147 (130–169)	0.417	145 (130–163)	140 (123–157)	145 (126–163)	150 (139–170)	0.005
Glucose, median (IQR), mmol/liter	6.97 (5.82–9.19)	5.60 (4.99–6.01)	6.91 (6.28–7.89)	9.80 (8.40–12.09)	<0.001	6.84 (5.73–8.56)	5.40 (4.94–5.84)	6.80 (6.30–7.34)	9.10 (7.97–13.00)	<0.001
HbA1C, median (IQR), %	5.90 (5.60–6.76)	5.90 (5.60–6.30)	5.80 (5.44–6.40)	6.20 (5.60–7.40)	0.032	5.90 (5.50–6.40)	5.80 (5.59–6.13)	5.80 (5.40–6.10)	6.00 (5.50–7.50)	0.052
SHR, median (IQR)	1.17 (1.01–1.36)	0.96 (0.85–1.00)	1.17 (1.11–1.23)	1.53 (1.37–1.72)		1.18 (0.98–1.40)	0.94 (0.85–0.99)	1.18 (1.11–1.23)	1.54 (1.38–1.72)	
Baseline NIHSS score, median (IQR)	16 (12–19)	16 (11–19)	15 (10–19)	17 (13–21)	0.034	16 (12–19)	16 (11–19)	16 (12–20)	15 (12–19)	0.207
Baseline ASPECTS, median (IQR)	8 (7–9)	8 (7–9)	8 (7–9)	8 (6–9)	0.079	8 (7–9)	7(7–9)	8 (7–9)	7 (6–9)	0.385
**Stroke etiology, No./Total No. (%)**					0.813					0.728
LAA	133/262 (50.8)	42/86 (48.8)	45/91 (49.5)	46/85 (54.1)		113/280 (40.4)	39/94 (41.5)	34/91 (31.4)	40/95 (42.1)	
CE	103/262 (39.3)	33/86 (38.4)	38/91 (41.8)	32/85 (37.6)		137/280 (48.9)	44/94 (46.8)	45/91 (49.5)	48/95 (50.5)	
Other causes	26/262 (9.9)	11/86 (12.8)	8/91 (8.8)	7/85 (8.2)		30/280 (10.7)	11/94 (11.7)	12/91 (13.2)	7/95 (7.4)	
**Occlusion sites, No./Total No. (%)**					0.924					0.591
ICA intracranial	54/262 (20.6)	16/86 (18.6)	20/91 (22.0)	18/85 (21.2)		64/280 (22.9)	26/94 (27.7)	19/91 (20.9)	19/95 (20.0)	
MCA-M1	168/262 (64.1)	55/86 (64.0)	57/91 (62.6)	56/85 (65.9)		178/280 (63.6)	54/94 (57.4)	59/91 (64.8)	65/95 (68.4)	
MCA-M2	40/262 (15.3)	15/86 (17.4)	14/91 (15.4)	11/85 (12.9)		38/280 (13.6)	14/94 (14.9)	13/91 (14.3)	11/95 (11.6)	

Onset to recanalization, median (IQR), min	480 (315–713)	499 (308–720)	510 (324–755)	450 (314–700)	0.738	454 (316–684)	453 (310–772)	460 (338–660)	453 (297–629)	0.782

Abbreviation: IQR, interquartile range; SBP, systolic blood pressure; HbA1C, glycated hemoglobin; SHR, stress hyperglycemia ratio; NIHSS, National Institutes of Health Stroke Scale; ASPECTS, Alberta Stroke Program Early Computed Tomography Score; LAA, large artery atherosclerosis; CE, cardio-embolism; ICA, internal carotid artery; MCA, middle cerebral artery.

1.3 eTable 3 Efficacy and safety outcomes according to SHR tertiles in different clinical treatment group

		Placebo			Tirofiban		
Outcomes	SHR levels	Events, n (%)	Unadjusted OR (95% CI)	Adjusted OR (95% CI)	Events, n (%)	Unadjusted OR (95% CI)	Adjusted OR (95% CI)
mRS 0-2							
	Tertile 1 (≤1.07)	46 (53.5)	Reference	Reference	57 (60.6)	Reference	Reference
	Tertile 2 (1.08-1.29)	42 (46.2)	0.75 (0.41–1.35)	0.67 (0.36–1.25)	49 (53.8)	0.76 (0.42–1.36)	0.83 (0.44–1.56)
	Tertile 3 (≥1.30)	33 (38.8)	0.55 (0.30–1.01)	0.65 (0.34–1.24)	29 (30.5)	0.29 (0.16–0.52)	0.36 (0.18–0.69)
mRS 0-1							
	Tertile 1 (≤1.07)	33 (38.4)	Reference	Reference	40 (42.6)	Reference	Reference
	Tertile 2 (1.08-1.29)	30 (33.0)	0.79 (0.43–1.46)	0.72 (0.38–1.37)	33 (36.3)	0.77 (0.43–1.39)	0.82 (0.43–1.55)
	Tertile 3 (≥1.30)	19 (22.4)	0.46 (0.24–0.90)	0.55 (0.27–1.11)	22 (23.2)	0.41 (0.22–0.76)	0.50 (0.25–0.99)
Mortality							
	Tertile 1 (≤1.07)	13 (15.1)	Reference	Reference	13 (13.8)	Reference	Reference
	Tertile 2 (1.08-1.29)	8 (8.8)	0.54 (0.21–1.38)	0.58 (0.22–1.52)	13 (14.3)	1.04 (0.45–2.38)	0.98 (0.41–2.34)
	Tertile 3 (≥1.30)	16 (18.8)	1.30 (0.58–2.91)	0.89 (0.37–2.11)	22 (23.2)	1.88 (0.88–4.00)	1.72 (0.75–3.99)
HBC_sICH							
	Tertile 1 (≤1.07)	2 (2.3)	Reference	Reference	8 (8.5)	Reference	Reference
	Tertile 2 (1.08-1.29)	3 (3.3)	1.43 (0.23–8.78)	1.50 (0.24–9.39)	7 (7.7)	0.90 (0.31–2.58)	0.95 (0.31–2.86)
	Tertile 3 (≥1.30)	10 (11.8)	5.60 (1.19–26.38)	5.49 (1.10–27.46)	9 (9.5)	1.13 (0.42–3.05)	1.15 (0.39–3.40)
Any ICH within 48h							
	Tertile 1 (≤1.07)	20 (23.3)	Reference	Reference	31 (33.0)	Reference	Reference
	Tertile 2 (1.08-1.29)	25 (27.5)	1.25 (0.63–2.47)	1.33 (0.66–2.69)	27 (29.7)	0.86 (0.46–1.60)	0.88 (0.45–1.70)
	Tertile 3 (≥1.30)	30 (35.3)	1.80 (0.92–3.52)	1.43 (0.70–2.93)	32 (33.7)	1.03 (0.56–1.89)	0.82 (0.42–1.63)

Adjusted for sex, diabetes, smoking, hypertension, hyperlipidemia, baseline NIHSS score, baseline ASPECTS.

Abbreviations: mRS, modified Rankin Scale; OR, odds ratio; CI, confidence interval; IQR, interquartile range; HBC_sICH, Heidelberg bleeding classification symptomatic intracranial hemorrhage; ICH, intracranial hemorrhage; ASPECTS, Alberta Stroke Program Early Computed Tomography Score.

2.Supplemental figures

2.1 eFigure 1: Flow diagram of the study

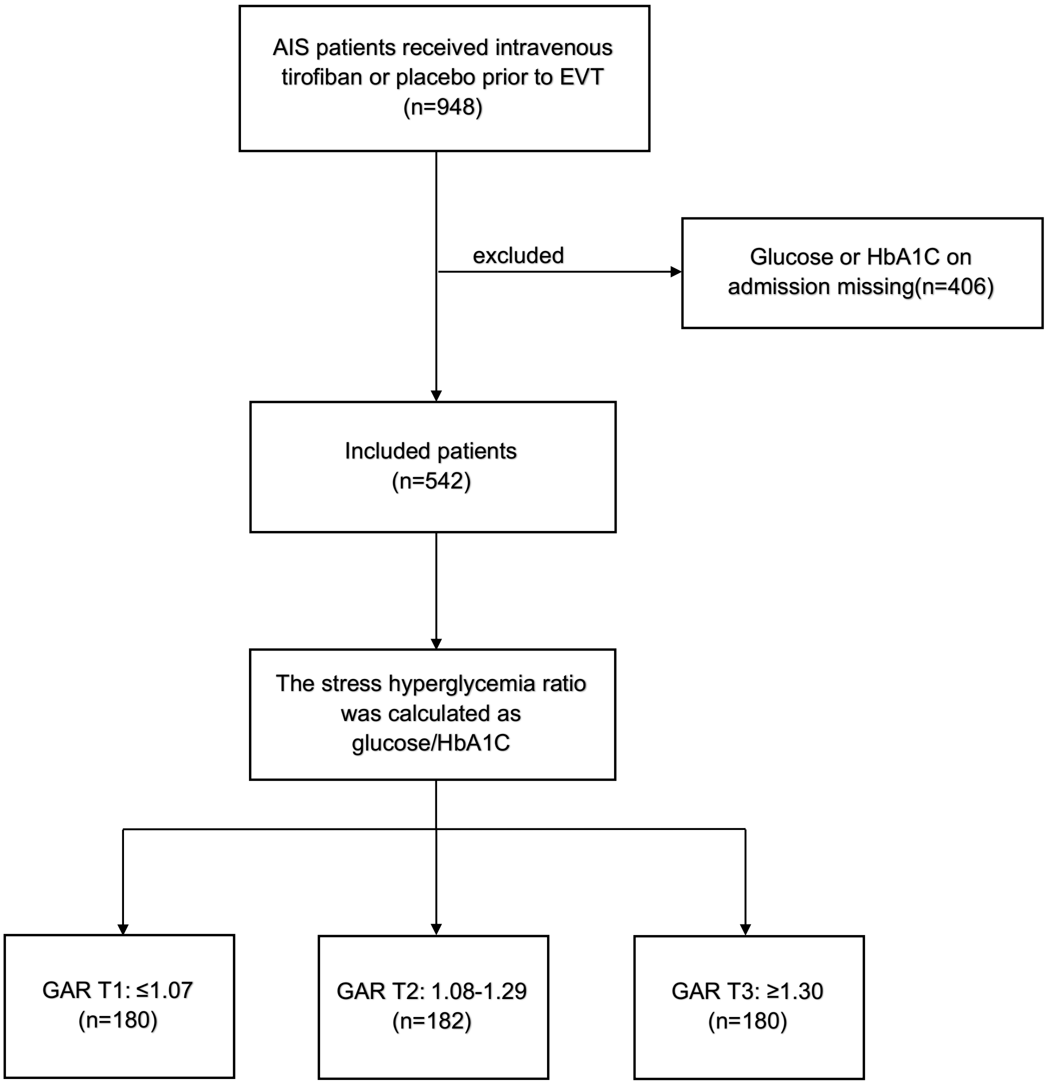

2.2 eFigure 2: The association between SHR and clinical outcomes

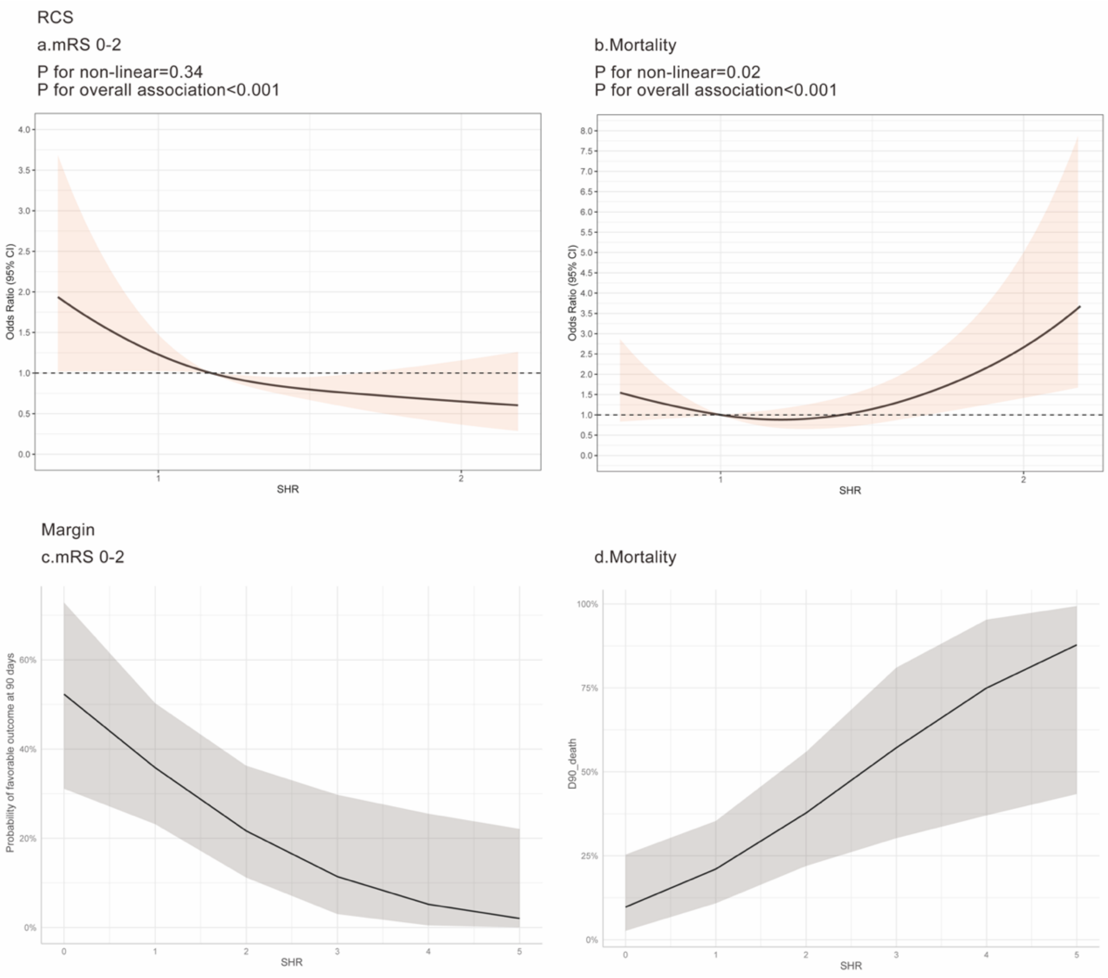

The association of SHR value with clinical outcomes in a restricted cubic spline model: (a) favorable functional outcome (mRS 0-2), (b) mortality within 90 days. The estimated probabilities of favorable functional outcome and mortality by SHR value among patients are presented in (c) and (d). Increased SHR value was associated with decreased estimated probabilities of favorable functional outcomes and increased estimated probabilities of mortality. Solid lines indicate estimated probabilities of outcomes; shaded areas, 95% CIs.

2.3 eFigure 3: Receiver operating characteristic (ROC) curves

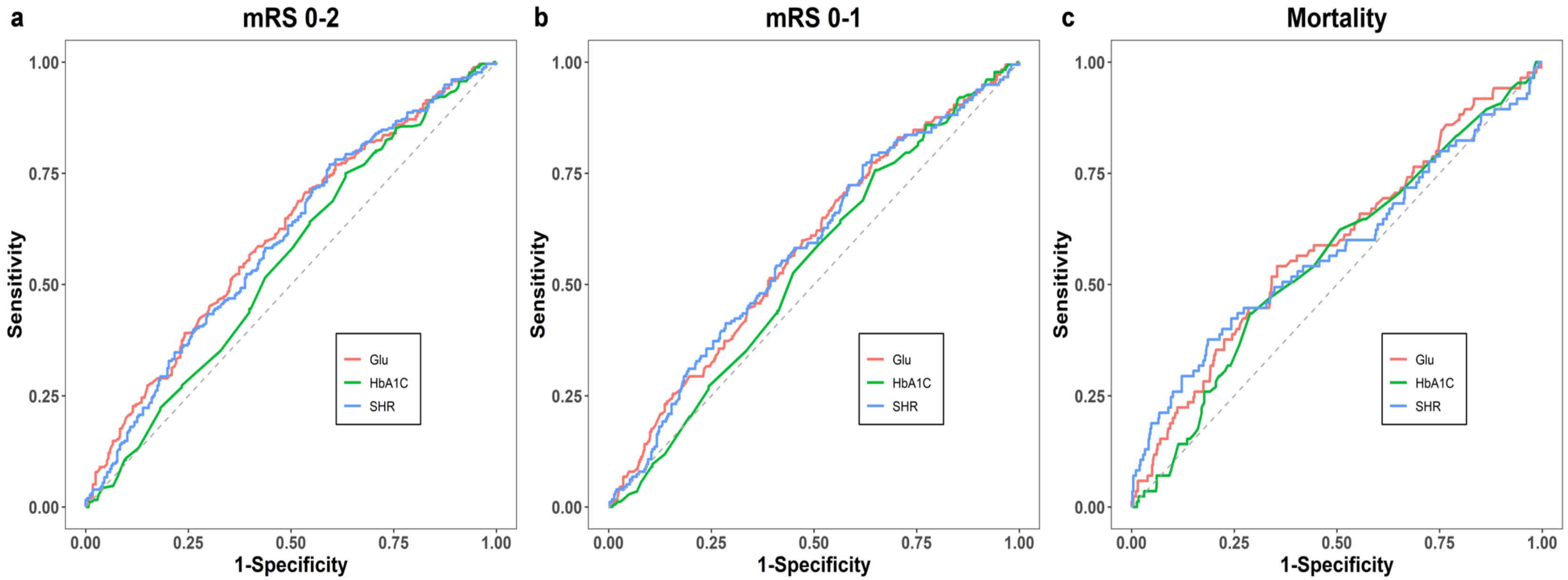

ROC curves for stress hyperglycemia ratio (SHR), glucose and glycosylated hemoglobin (HbA1C) with clinical outcomes including mRS 0-2 (a), mRS 0-1(b) and mortality (c) as endpoint.
